# Side-effects of intravenously *versus* intramuscularly oxytocin for *postpartum* hemorrhage: a systematic review and meta-analysis of randomized controlled trials

**DOI:** 10.3389/fphar.2023.1273771

**Published:** 2023-12-22

**Authors:** Wen Ai, Yanfei Zeng, Manhua Zhen, Li Lao, Yubo Ma, Li Liu, Yinghui Zhang

**Affiliations:** ^1^ Department of Obstetrics and Gynecology, Foshan Fosun Chancheng Hospital, Foshan, Guangdong, China; ^2^ Department of Epidemiology and Biostatistics, School of Public Health, Anhui Medical University, Hefei, Anhui, China; ^3^ Department of Library, The First Affiliated Hospital, College of Medicine, Zhejiang University, Hangzhou, Zhejiang, China

**Keywords:** *Postartum* hemorrhage, oxytocin, side-effects, route of administration, randomized controlled trial

## Abstract

**Background:** Oxytocin is the gold standard uterotonic agent for prevention of *postpartum* hemorrhage. However, there is no consensus with clear evidence about the side-effects of oxytocin administered intravenously or intramuscularly for management of the third stage of labor. We conducted a systematic review and meta-analysis of randomized controlled trials to evaluate the side-effects of intravenously or intramuscularly oxytocin for preventing *postpartum* hemorrhage in the third stage of labor.

**Methods:** Six representative databases were searched from the inception to July 2023. Randomized controlled trials which explored the intravenously and intramuscularly oxytocin and provided at least one side-effect were included. Statistical analysis included random or fixed-effect meta-analyses using relative risk.

**Results:** Nine studies included, involving 8,295 participants. Ten types of side-effects were reported. There was no statistical difference in hypotension (RR = 1.01, 95%CI = 0.88–1.15), anemia (0.98, 0.83–1.15), tachycardia (0.90, 0.69–1.17), shivering (0.90, 0.69–1.17), headache (0.86, 0.31–2.37), nausea (0.70, 0.20–2.42), vomiting (0.97, 0.26–3.58), uvular edema (0.82, 0.23–2.91), diarrhea (0.97, 0.26–3.58), and fever (0.97, 0.26–3.58) between intravenously or intramuscularly groups.

**Conclusion:** There are no significant differences of side-effects between intravenously and intramuscularly administration of oxytocin for preventing *postpartum* hemorrhage in the third labor.

**Systematic Review Registration:** https://www.crd.york.ac.uk/PROSPERO/display_record.php?RecordID=407571.

## Introduction

*Postartum* hemorrhage is one of the leading causes of pregnancy-related mortality and severe morbidity, including blood transfusion, prolonged hospital stay and hysterectomy, and nearly a quarter of all maternal deaths are associated with *postpartum* hemorrhage (Say et al., 2014; Rozenberg et al., 2023). The initial prevention and treatment *postpartum* hemorrhage are mainly involved uterine massage, medical management, and uterotonic drugs (Committee on Practice, 2017). For many years, oxytocin remains the preferred choice pharmacologic agent that plays a central role in the prevention of *postpartum* hemorrhage (Weeks, 2021). The value of widely used uterotonic pharmaceuticals (oxytocin, ergometrine, and misoprostol) in the third stage of pregnancy is currently widely acknowledged, however, oxytocin is still the gold standard uterotonic agent and the first choice for prevention of *postpartum* hemorrhage, because it has similar efficacy, no major contraindications, and is inexpensive, compared with other available options (Jaffer et al., 2022; Jones et al., 2023). Normally, oxytocin can be administered intravenously or intramuscularly (Adnan et al., 2018; Charles et al., 2019). It has been widely assumed that both routes are highly effective, and international guidelines, including American College of Obstetricians and Gynecologists and the World Health Organizaiton, currently recommend both routes equally (Organization, 2012; Committee on Practice, 2017).

Each route of oxytocin has potential benefits and side-effects. In addition to the therapeutic effects, side-effects are an essential attribute to take into account in clinical practice (Ai et al., 2021). A recent systematic review and meta-analysis including 61 randomized controlled trials reported that compared with other uterotonic agent(s), oxytocin had been linked to considerably fewer instances of diarrhea, fever, and shivering events and was not increasing the probability of other side-effects during the third stage of labor (Zeng et al., 2022). Currently, available systematic reviews and meta-analyses have all explored the effectiveness of oxytocin administered intravenously or intramuscularly for management of the third stage of labor (Ebada et al., 2020; Wu et al., 2020; Torloni et al., 2021; Behuria et al., 2023). However, there is no consensus with clear evidence among the clinicians about the side-effects.

Thus, we conducted a systematic review and meta-analysis of randomized controlled trials to evaluate the side-effects of intravenously or intramuscularly oxytocin for preventing *postpartum* hemorrhage in the third stage of labor, wishing to contribute in helping guide clinical practice.

## Methods

We reported this study in accordance with the PRISMA Statement and Checklist (Page et al., 2021; Fan et al., 2023). The identifying number is CRD42023407571 in the International Prospective Register of Systematic Reviews (PROSPERO).

### Search strategy

The PubMed, Embase, Web of Science, ClinicalTrials.gov, Elsevier ScienceDirect, and the Cochrane Library databases were used to search from inception to 31 July 2023. An experienced medical librarian conducted the search strategy using the keywords: “oxytocin” and “*postpartum* hemorrhage”. Supplementary file 1 provided the detailed search strategy.

### Eligibility criteria

Randomized controlled trials which explored the oxytocin for *postpartum* hemorrhage were selected. The inclusion criteria were compared intravenously with intramuscularly and provided adverse events or side-effect data. Participants with anticoagulant therapy, bleeding disorders, or cardiac illness were not included. Quasi-randomised studies, letters to the editor, review, and corresponding were all excluded.

### Data extraction

Microsoft excel standardized data extraction procedure was using to extract the primary data by two authors independently. Primary data collection included: the name of the first author, publication year, study period, study country, registration status, funding situation, sample size, delivery mode, risk of *postpartum* hemorrhage, dose and route of oxytocin, age of pregnancy women, the type and number of side-effects, in each included randomized controlled trial. Discussion resolved any disagreements between the two authors for the data extraction. If needed, authors contacted the included corresponding author to obtain the missing data.

### Risk of bias assessment

Two independently authors were assessed the methodological quality using the Cochrane handbook (Cumpston et al., 2019; Ai et al., 2021; Zeng et al., 2022). According to standard, three levels, high-, unclear-, or low-risk, were defined of each quality item. At the same time, high-, moderate-, or low-quality, was assigned to each included randomized controlled trial. Regardless of the results of other items, if random sequence generation or allocation concealment was defined as high-risk of bias, the studies were graded as low-quality; if random sequence generation and allocation concealment were all defined as low-risk of bias, meanwhile, all other items were not defined as high-risk of bias, the studies were graded as high-quality; other included studies were graded as unclear-quality.

### Data analysis

According to the Cochrane Handbook, if there is zero events in one group, 0.5 was added to each cell in the fourfold table. The results were measured by risk ratios (RRs) with 95% confidence intervals (CIs). If there was heterogeneity among included studes, the random-effects was performed to calculate the results. Otherwise, fixed-effect was used to calculate the results. *I* ^*2*^ statistic was used to calculate the statistical heterogeneity. Begg and Egger tests were used to evaluate the publications bias. Funnel plot was also given to display the symmentry of the included studies. We used review Manager 5.4, and R software 3.2.2 to conduct the meta-analysis.

## Results

### Study selection and characteristics

**FIGURE 1:**
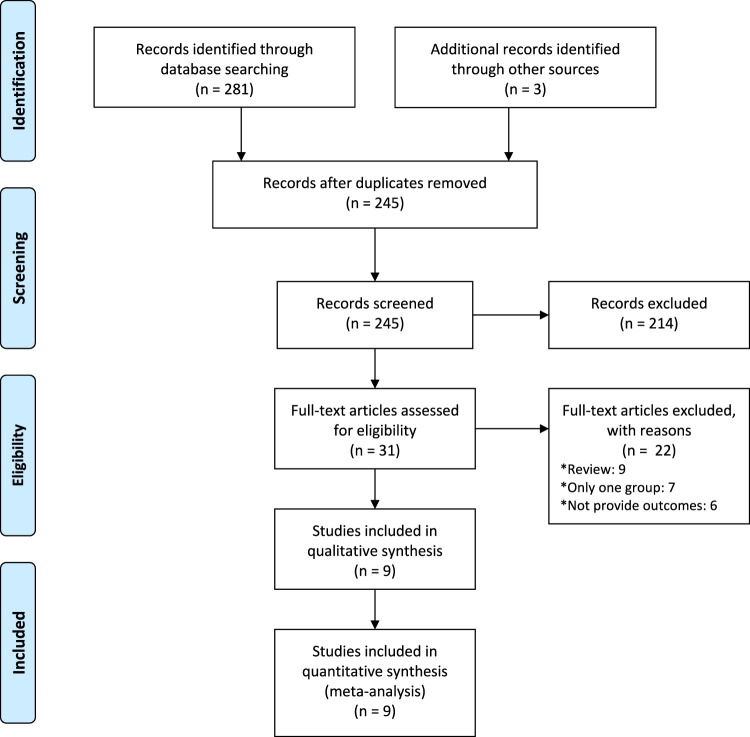
Flow diagram of the study selection process for this systematic review and meta-analysis.

A total of 1,420 records were yielded throughout the database search. We assessed 338 full-texts for eligibility after removing duplicates. Overall, nine studies met inclusion criteria and included, involving 8,295 participants (Figure 1) (Oguz Orhan et al., 2014; Sangkomkamhang and Kruangpatee, 2015; Dagdeviren et al., 2016; Neri-Mejia and Pedraza-Aviles, 2016; Devi and Bhatnagar, 2017; Adnan et al., 2018; Charles et al., 2019; Durocher et al., 2019; Ashwal et al., 2022).

The characteristics of the included randomized controlled trials were displayed in Table 1. The published year is from 2014 to 2022. The median number of sample sizes per study was 450 (range, 43-4,913). Two studies were from Turkey, (Oguz Orhan et al., 2014; Dagdeviren et al., 2016), and other seven studies are from Thailand, (Sangkomkamhang and Kruangpatee, 2015), Mexico, (Neri-Mejia and Pedraza-Aviles, 2016), India, (Devi and Bhatnagar, 2017), Ireland, (Adnan et al., 2018), Eygp, (Charles et al., 2019), Argentina, (Durocher et al., 2019), and Irael, (Ashwal et al., 2022), respectively. All of participants underwent vaginal birth and used standard dose (10 iu). Five studies provided the trial registration number, and three studies stated their funding. Totally, ten types of side-effects, including hypotension, anemia, tachycardia, shivering, headache, nausea, vomiting, uvular edema, diarrhea, and fever, were reported in this study (Table 1).

**TABLE 1:** General characteristics of included studies.

First author	Publish year	Enrolment period	Trail no.	Funded	Country	Risk for PPH	Delivery mode	Interventions (sample size; dose; adm)	Side effects
Ashwal E	2022	2014.4–2015.9	NCT02319707	NA	Israel	L	VD	57, 10 iu IV, vs. 61, 10 iu IM	Any side effect
Durocher J	2019	2016.12–2017.9	NA	The Bill and Melinda Gates Foundation	Argentina	L	VD	239, 10 iu IV vs. 241, 10 iu IM	Nausea
									Vomiting
									Diarrhoea
									Fever
									Shivering
									Headache
									Hypotension
									Tachycardia
Charles D	2019	2014.4–2015.9	NCT01914419	The Bill and Melinda Gates Foundation	Egypt	L	VD	2,809, 10 iu IV vs. 2,104, 10 iu IM	Anemia
									Hypotension
Adnan N	2018	2016.1–2017.12	ISRCTN14718882	Trinity College, University of Dublin, and Coombe Women and Infants University Hospital	Ireland	L	VD	517, 10 iu IV vs. 518, 10 iu IM	Any side effect
									Nausea
									Vomiting
									Shivering
									Headache
									Hypotension
									Tachycardia
Devi AM	2017	2015.4–2016.9	NA	NA	India	L	VD	200, 10 iu, IV vs. 200, 10 iu, IM	NA
Neri-Mejia M	2016	2015.8–2015.12	NA	NA	Mexico	L	VD	21, 10 iu, IV vs. 22, 10 iu, IM	Hypotension
Dagdeviren H	2016	2014.2–2015.3	NCT02080104	NA	Turkey	L	VD	128, 10 iu, IV vs. 128, 10 iu, IM	Shivering
									Nausea
									Vomiting
									Pyrexia
									Tachycardia
Sangkomkamhang U	2015	2012.2–2012.6	NA	NA	Thailand	L	VD	225, 10 iu, IV vs. 225 10 iu, IM	Serious side effects
Oguz Orhan E	2014	2010.1–2010.10	NCT01954186	NA	Turkey	L	VD	300, 10 iu IV vs. 300, 10 iu IM	Uvular Edema

L, low risk for PPH; NA, none; PPH, *postpartum* hemorrhage; VD, vaginal birth.

### Risk of bias

**FIGURE 2:**
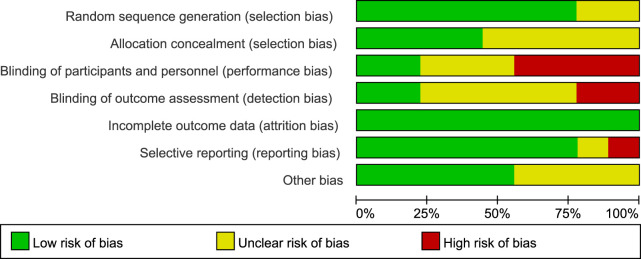
Proportions of articles that met each criterion for risk of bias across the 9 included randomized controlled trials.

**FIGURE 3:**
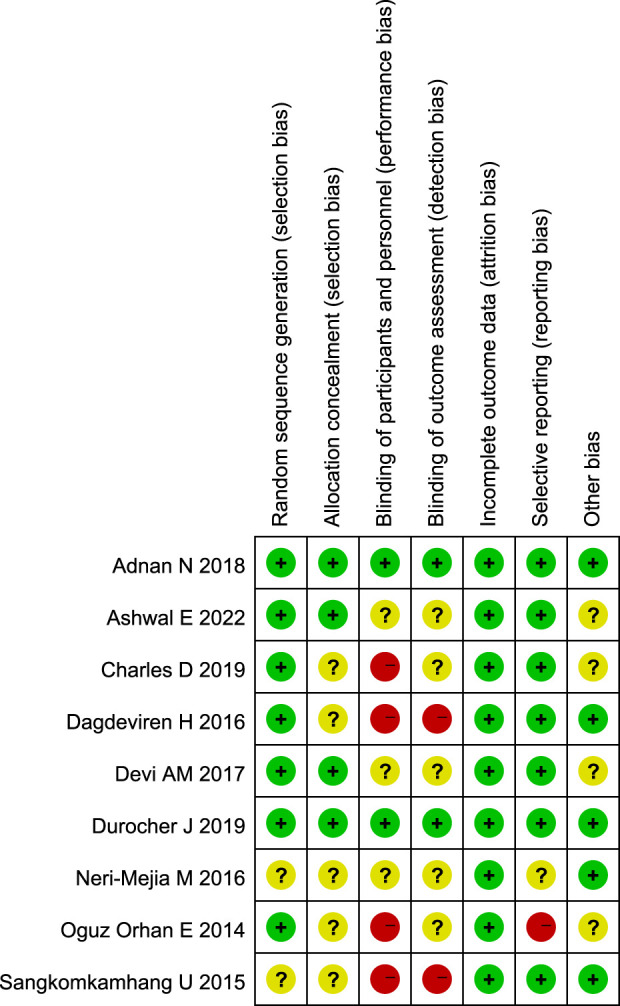
Results of the risk of bias for 9 included randomized controlled trials. Green means low risk; yellow means unclear risk; red means high risk.

The detailed risk of bias was provided in Figures 2, 3, respectively. Of the total 63 items, there are 36 low-risk items 20 unclear-risk items, and 7 high-risk item. Seven studies were randomized; two were adequately concealed allocation and double-blind; 35 trials blinded outcome assessors; nine were described the incomplete outcome data or provided the complete outcome data. Overall, two studies classified to high quality; four studies classified to low quality; and three studies classified to moderate quality.

### Outcomes

There was no statistical difference in hypotension (RR = 1.01, 95%CI = 0.88–1.15), anemia (0.98, 0.83–1.15), tachycardia (0.90, 0.69–1.17), shivering (0.90, 0.69–1.17), headache (0.86, 0.31–2.37), nausea (0.70, 0.20–2.42), vomiting (0.97, 0.26–3.58), uvular edema (0.82, 0.23–2.91), diarrhea (0.97, 0.26–3.58), and fever (0.97, 0.26–3.58) between intravenously or intramuscularly groups (Supplementary Figures S1–S10).

### Publication bias

Funnel plots observed symmentry for the side-effects (Supplementary Figures S11–S20). Meanwhile, Begg and Egger tests also demonstrated that there was no publication bias for side-effects.

## Discussion

In this meta-analysis, we attempted to evaluate the side-effects of intravenously and intramuscularly oxytocin by studying nine randomized controlled trials with over 8,000 participants. Our analysis unequivocally revealed intravenously compared with intramuscularly oxytocin administration may result in little to no difference on the incidence of side-effects.

Today, several uterotonic agents are recommended for *postpartum* hemorrhage prophylaxis, including oxytocin, misoprosol, ergometrine, and methylergonovine, but oxytocin is still the preferred choice compared with other available options (Committee on Practice, 2017). In addition to effects, there are several aspects to take into account when choosing how to administer oxytocin, such as the personal preferences of the women, the supplies available, and the level of expertise of the giver. There is currently inadequate information to support the side-effects of intravenously *versus* intramuscularly oxytocin treatment.

There are some differences in advantages and disadvantages between intravenously and intramuscularly oxytocin. Intravenously oxytocin mainly reflects the clinical advantages. The response is almost instantaneous, and the plasma oxytocin levels could reach the peak quickly, reaching a stable concentration at half an hour (Gibbens et al., 1972; Adnan et al., 2018; Charles et al., 2019). Because of the quickly, cardiovascular side-effects, such as, hypotension, tachycardia, electrocardiographic changes, are the most commonly reported for intravenously oxytocin for preventing *postpartum* hemorrhage (Choy et al., 2002; Davies et al., 2005; Thomas et al., 2007). Meanwhile, other side-effects, including chest pain, pulmonary edema, flushing, headache, nausea, vomiting, severe water intoxication, and convulsion, are also been mentioned in several clinical trials of intravenously oxytocin (Dagdeviren et al., 2016; Adnan et al., 2018; Charles et al., 2019).

Because of less equipment of administer and requiring relatively lesser skills, intramuscularly oxytocin appears to have practical advantages, making it more widely option, especially in areas with underdeveloped medical resources (Rashid et al., 2009; Oladapo et al., 2018). Within 3–7 min, intramuscular route produces a uterotonic response that continues for thirty to 60 minutes (Adnan et al., 2018). By contrast, there is little data on the side-effects of intramuscularly oxytocin for preventing *postpartum* hemorrhage. This may be because there are few side-effects that are clinically important. Of course, a small number of clinical side-effects, such as hypotension, tachycardia, nausea, shivering, and headache, have also been noted with intramuscularly oxytocin for preventing *postpartum* hemorrhage (Adnan et al., 2018; Charles et al., 2019; Durocher et al., 2019). Significantly, abscess and pain at the injection site, which are common side-effects of any intramuscular injection, also occur with intramuscularly oxytocin, especially if safety procedures are not followed (Dagdeviren et al., 2016). In our study, there was no significant difference in the incidence of side-effects between the two groups.

As far as we are concerned, this is the first systematic review and meta-analysis focused on evaluating the side-effects of intravenously and intramuscularly oxytocin for preventing *postpartum* hemorrhage. The limitations include the studies enrolled were small sample-sized, with high risks of bias, which made the systemic review with limited clinical relevance. Most of the enrolled studies were conducted in developing countries, and the variation in side-effect reporting across trials, then the generalizability of the results and conclusions were limited. In addition, trials varied slightly in some diagnoses side-effects, which could have influenced our findings. We advise future studies to use uniform diagnostic standards in order to strengthen the evidence.

## Conclusion

In summary, the study found that there are no significant differences of side-effects between intravenously and intramuscularly administration of oxytocin for preventing *postpartum* hemorrhage in the third labor. To strengthen the evidence base, we look forward to large-scale trials to further explore this question.

## Data Availability

The original contributions presented in the study are included in the article/Supplementary Material, further inquiries can be directed to the corresponding author.

## Abbreviations

CI, confidence intervals; IM, intramuscularly; IV, intravenously; PPH, *postpartum* hemorrhage; RR, risk ratio.
