# Supplementary material for: Side-effects of intravenously versus intramuscularly oxytocin for postpartum hemorrhage: a systematic review and meta-analysis of randomized controlled trials
Source: Front Pharmacol. 2023 Dec 22;14:1273771. doi: 10.3389/fphar.2023.1273771 (PMC10770861; doi:10.3389/fphar.2023.1273771)
Supplement: Supplementary file 1 [file Table2.DOC]

**Supplementary files**

Supplementary file 1. Appendix_1_Search strategies (P1)

Supplementary file 2. The results of forest in each side-effects (P2-P11)

Supplementary file 3. The results of funnel plots in each side-effects (P12-P21)

**Supplementary 1.** Search strategy

((randomized controlled trial[Publication Type]) OR (controlled clinical trial[Publication Type]) OR randomized[Title/Abstract] OR placebo[Title/Abstract] OR randomly[Title/Abstract] OR trial[Title/Abstract] OR groups[Title/Abstract]) AND ((third stage[All Fields]) AND (labor[All Fields] OR labour[All Fields]) AND Oxytocin[All Fields] AND (haemorrhage[All Fields] OR hemorrhage[All Fields]) AND postpartum[All Fields])

**Supplementary file 2.** The results of forest in each side-effects


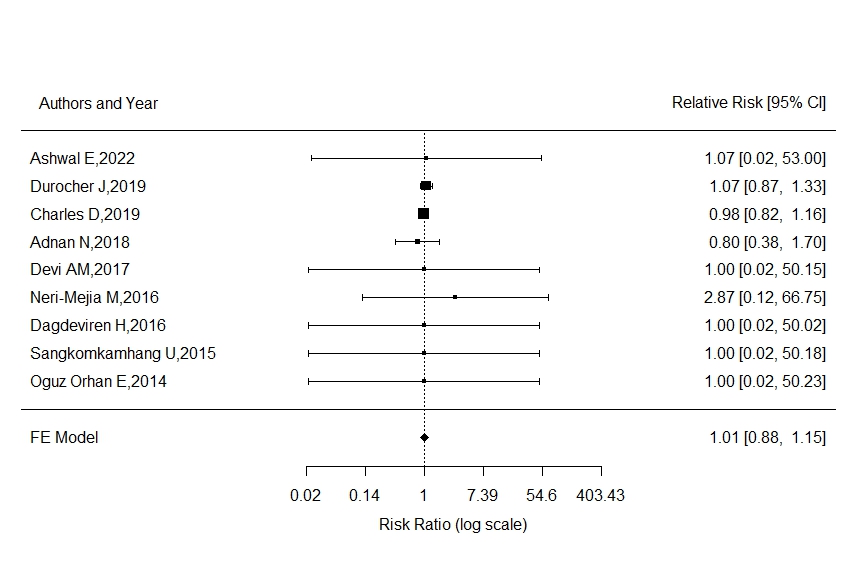


Figure S1. Meta-analysis of the association between hypotension and oxytocin routes.


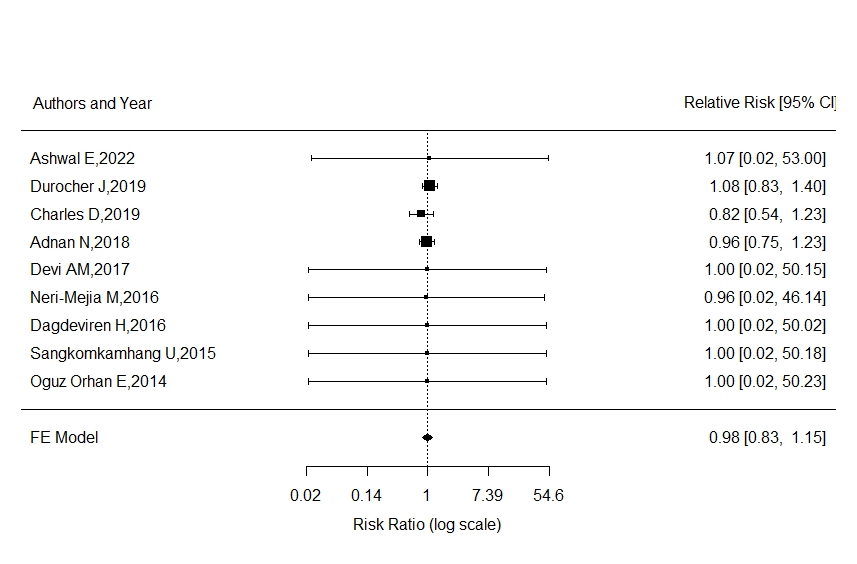


Figure S2. Meta-analysis of the association between anemia and oxytocin routes.


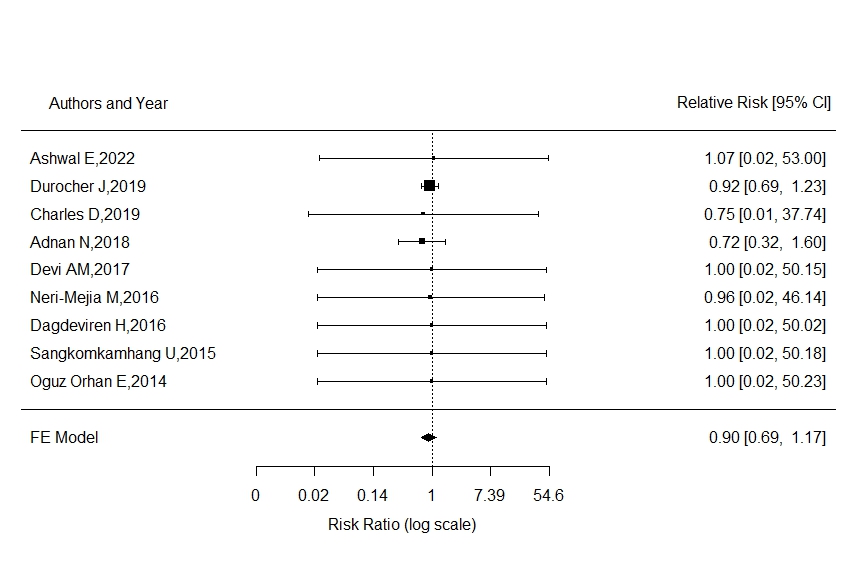


Figure S3. Meta-analysis of the association between tachycardia and oxytocin routes.


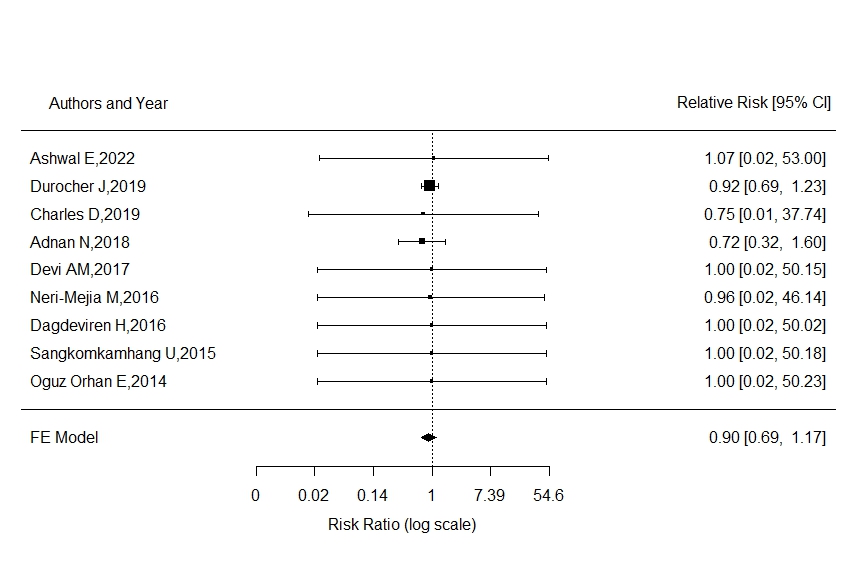


Figure S4. Meta-analysis of the association between shivering and oxytocin routes.


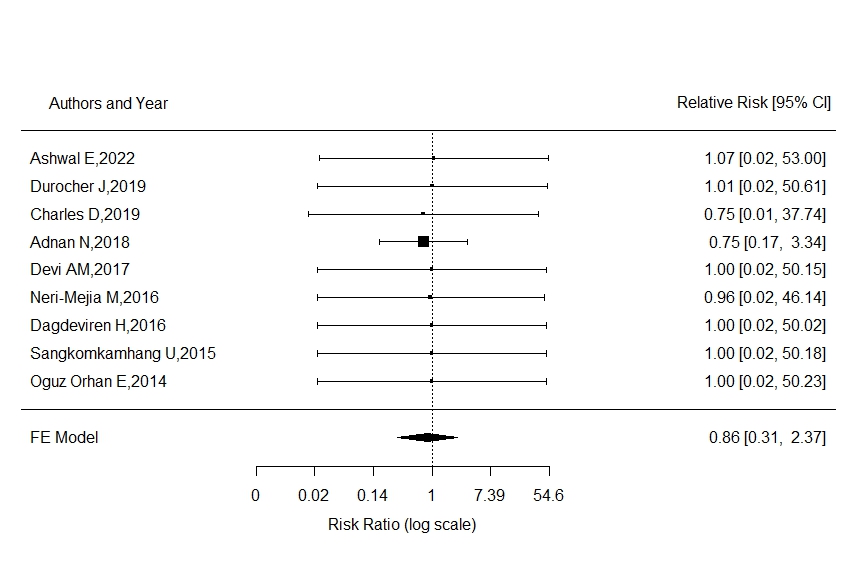


Figure S5. Meta-analysis of the association between headache and oxytocin routes.


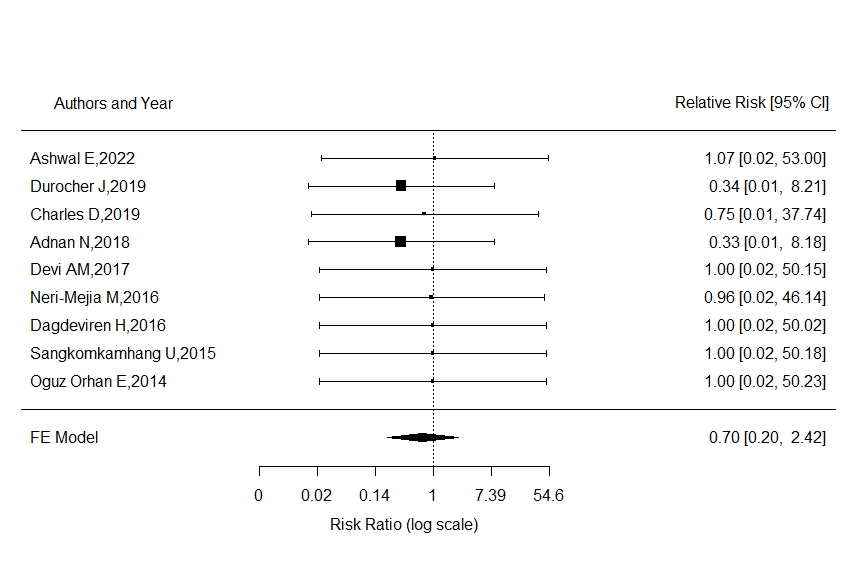


Figure S6. Meta-analysis of the association between nausea and oxytocin routes.


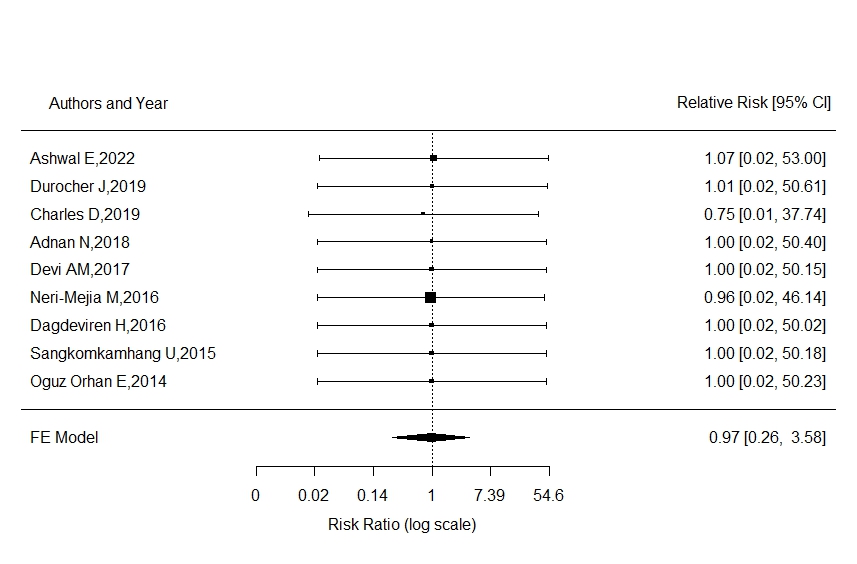


Figure S7. Meta-analysis of the association between vomiting and oxytocin routes.


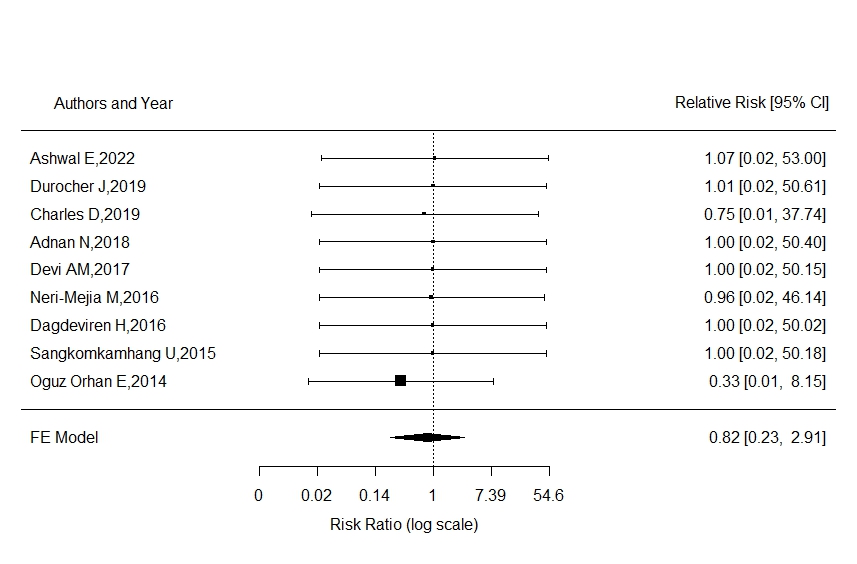


Figure S8. Meta-analysis of the association between uvular edema and oxytocin routes.


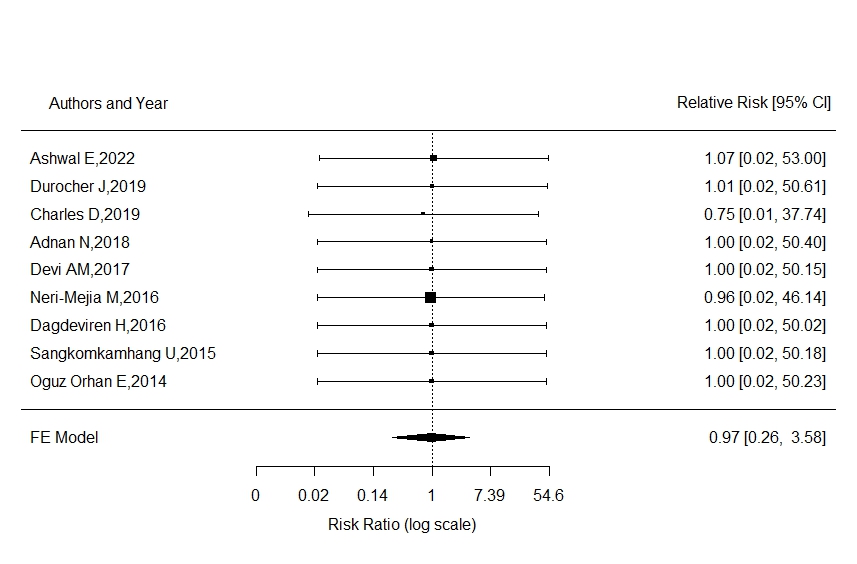


Figure S9. Meta-analysis of the association between diarrhea and oxytocin routes.


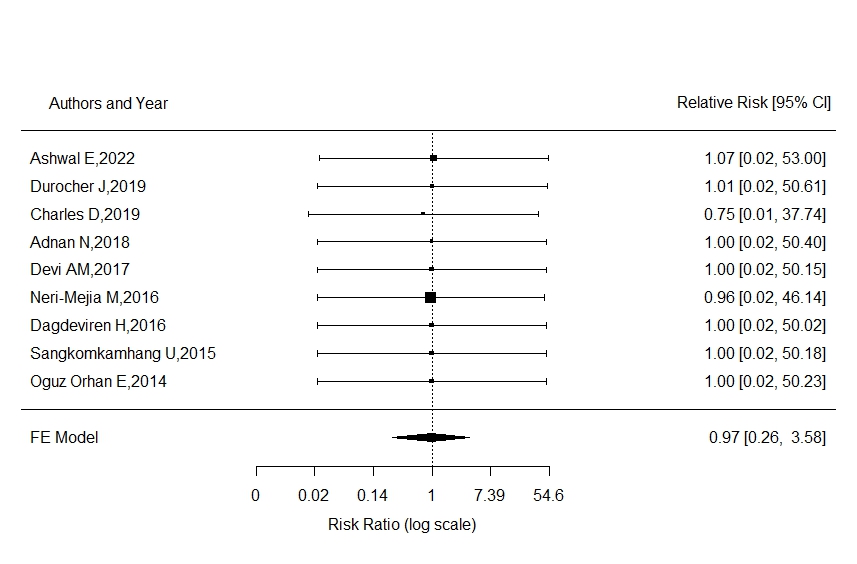


Figure S10. Meta-analysis of the association between fever and oxytocin routes.

**Supplementary file 3.** The results of funnel plots in each side-effects


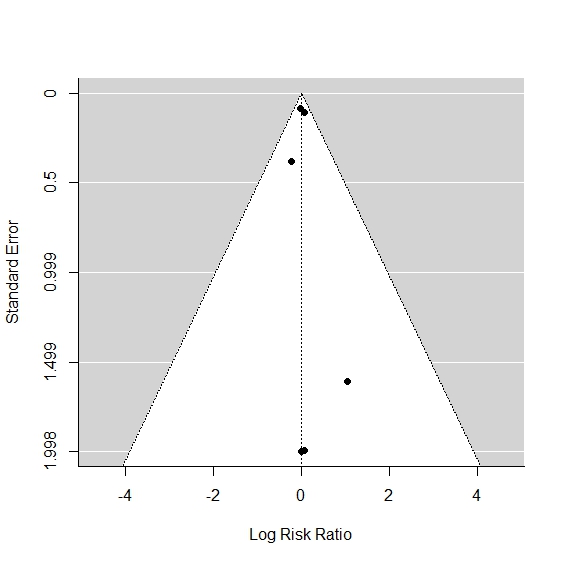


Figure S11. Funnel plot of included studies for side-effects. (**hypotension**)


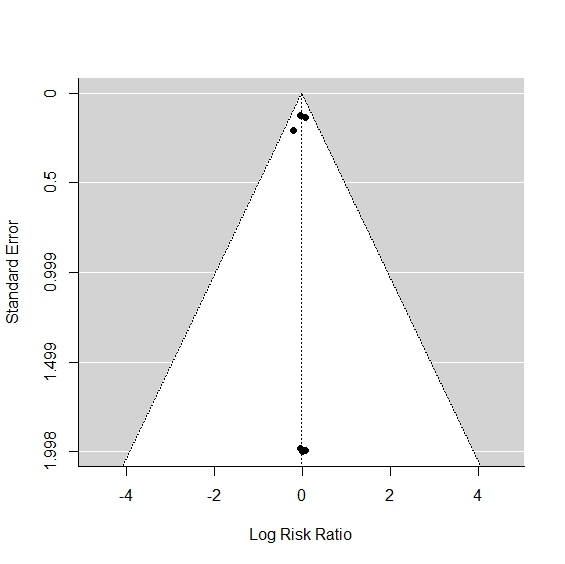


Figure S12. Funnel plot of included studies for side-effects. (**anemia**)


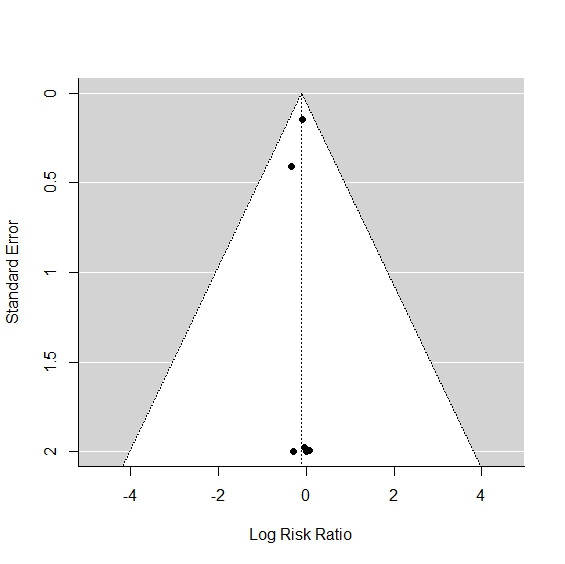


Figure S13. Funnel plot of included studies for side-effects. (**tachycardia**)


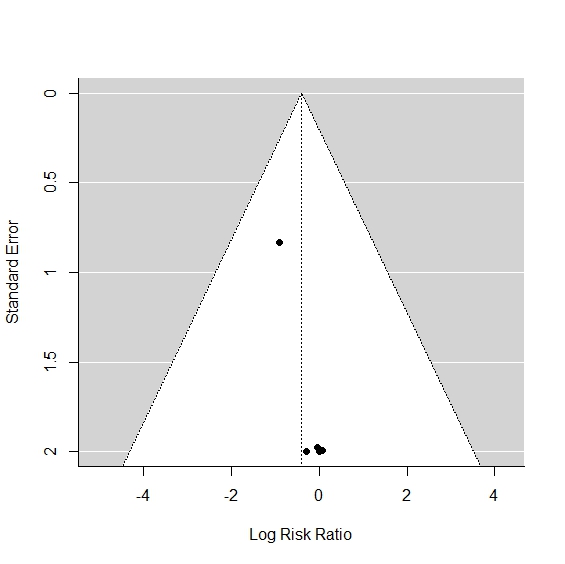


Figure S14. Funnel plot of included studies for side-effects. (**shivering**)


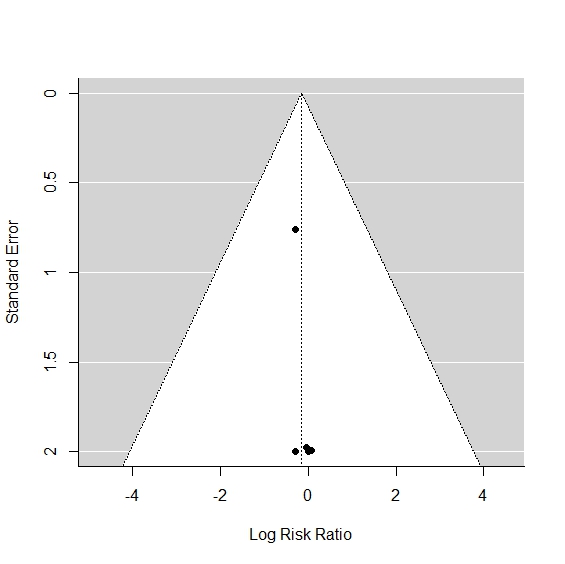


Figure S15. Funnel plot of included studies for side-effects. (**headache**)


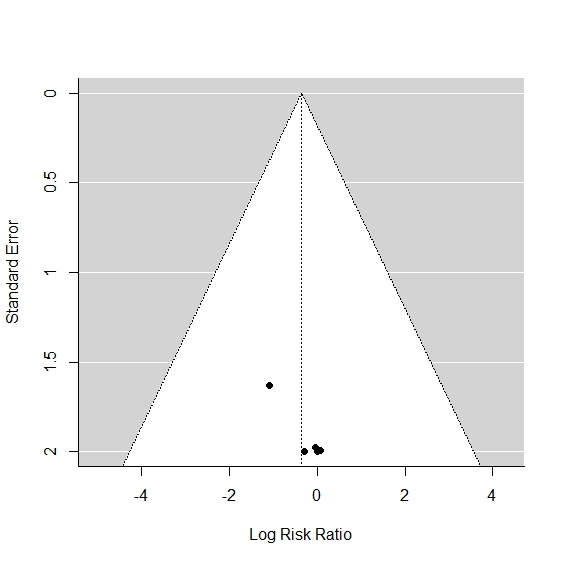


Figure S16. Funnel plot of included studies for side-effects. (**nausea**)


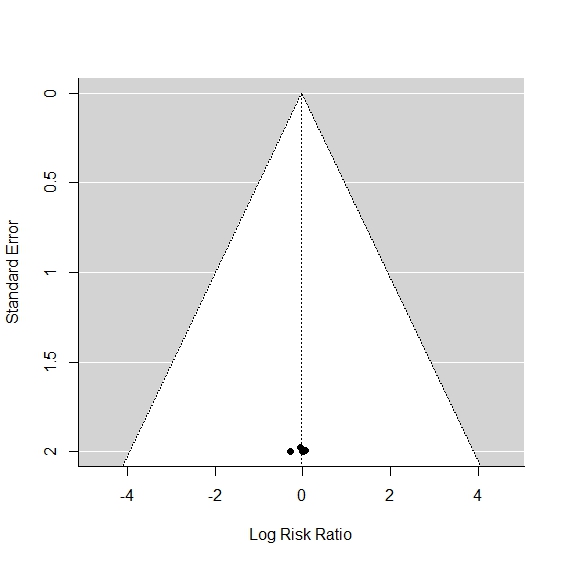


Figure S17. Funnel plot of included studies for side-effects. (**vomiting**)


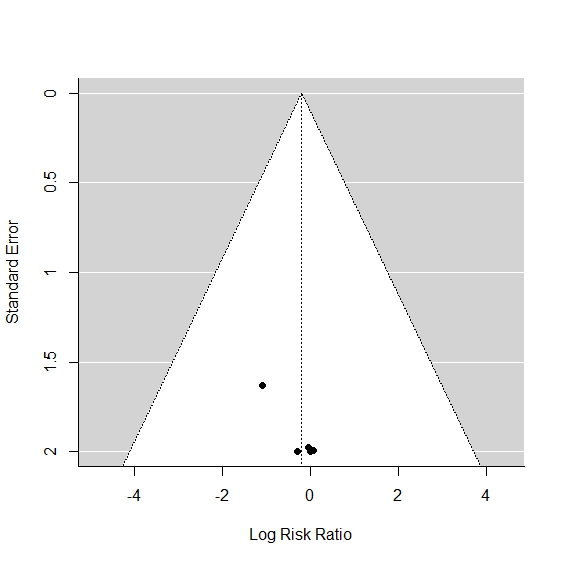


Figure S18. Funnel plot of included studies for side-effects. (**uvular edema**)


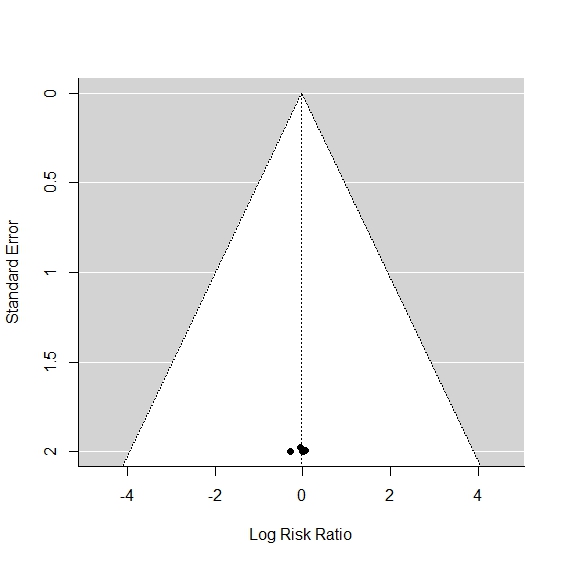


Figure S19. Funnel plot of included studies for side-effects. (**diarrhea**)


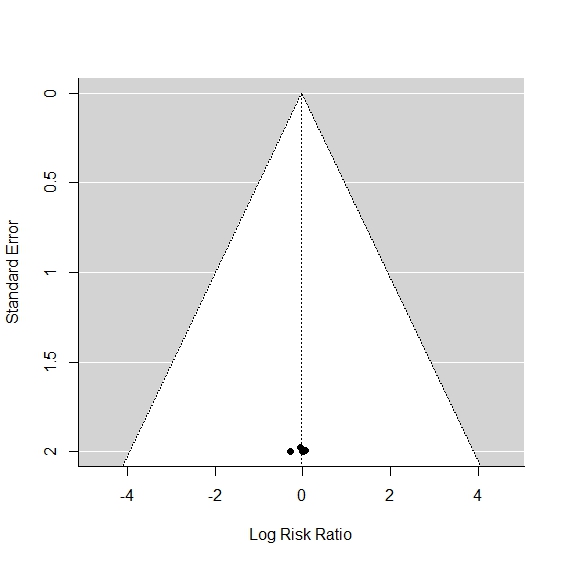


Figure S20. Funnel plot of included studies for side-effects. (**fever**)
